# BIM-Ken: Identifying Disease-Related miRNA Biomarkers Based on Knowledge-Enhanced Bio-Network

**DOI:** 10.3390/genes16080902

**Published:** 2025-07-28

**Authors:** Yanhui Zhang, Kunjie Dong, Wenli Sun, Zhenbo Gao, Jianjun Zhang, Xiaohui Lin

**Affiliations:** 1 School of Computer Science and Technology, Dalian University of Technology, Dalian 116024, China; 2 Department of Gastric Surgery, Cancer Hospital of Dalian University of Technology (Liaoning Cancer Hospital & Institute), Shenyang 110042, China

**Keywords:** miRNA biomarker identification, bio-networks, miRNA-disease associations, omics data analysis, graph auto-encoder

## Abstract

The identification of microRNA (miRNA) biomarkers is crucial in advancing disease research and improving diagnostic precision. Network-based analysis methods are powerful for identifying disease-related biomarkers. However, it is a challenge to generate a robust molecular network that can accurately reflect miRNA interactions and define reliable miRNA biomarkers. To tackle this issue, we propose a disease-related miRNA biomarker identification method based on the knowledge-enhanced bio-network (BIM-Ken) by combining the miRNA expression data and prior knowledge. BIM-Ken constructs the miRNA cooperation network by examining the miRNA interactions based on the miRNA expression data, which contains characteristics about the specific disease, and the information of the network nodes (miRNAs) is enriched by miRNA knowledge (i.e., miRNA-disease associations) from databases. Further, BIM-Ken optimizes the miRNA cooperation network using the well-designed GAE (graph auto-encoder). We improve the loss function by introducing the functional consistency and the difference prompt, so as to facilitate the optimized network to keep the intrinsically important characteristics of the miRNA data about the specific disease and the prior knowledge. The experimental results on the public datasets showed the superiority of BIM-Ken in classification. Subsequently, BIM-Ken was applied to analyze renal cell carcinoma data, and the defined key modules demonstrated involvement in the cancer-related pathways with good discrimination ability.

## 1. Introduction

MicroRNAs (miRNAs) are a class of small non-coding RNAs, and they are about 22 nucleotides in length [1]. Numerous studies have shown that miRNAs play critical roles in various biological processes by targeting specific mRNA and regulating gene expression [2,3], including cell growth, cell proliferation, and immune reaction. The abnormal expression of miRNAs may affect these biological processes and lead to various diseases [4], and many miRNAs can act as tumor suppressors and oncogenes [3]. Therefore, miRNAs can be used as potential disease biomarkers [5]. Identifying the miRNA biomarkers is of great value in elucidating disease mechanisms, improving disease prognosis, and refining therapeutic strategies.

Feature selection methods have been investigated and applied to analyze the miRNA expression data and identify the discriminative miRNAs as the potential biomarkers for diseases [6,7]. Fold change and *t*-test are commonly used methods. However, these methods focus on miRNA distinguishing ability without considering miRNA interactions, and the emerging evidence has shown that miRNA interactions play an important role in the occurrence and development of diseases [8]; considering the miRNA interactions can help define more accurate biomarkers reflecting physiological and pathological changes [9].

Network-based data analysis approaches provide in-depth insights into the molecular (e.g., gene, metabolite, miRNA) interactions at the system level [10]. The network is depicted as a graph structure, where nodes represent molecules and edges reflect the interactions between them [11]. In terms of miRNA networks, correlations are commonly used for network construction. Li et al. [12] used the Spearman correlation coefficient to construct the relevant connection network, and the consistency and differences of relevant connection networks under different states were analyzed to define the key miRNAs related to early gastric cancer. Zhang et al. [13] adopted the Pearson correlation coefficient (PCC) to establish the weighted gene co-expression network, and identified the modules using the topological overlap measure, which has been applied to miRNA co-expression network analysis [14]. These methods use statistical techniques to construct the miRNA networks based on the miRNA expression data, reflecting the miRNA interactions in a specific disease.

Meantime, there are publicly available knowledge bases, such as miRTarBase, HMDD (Human MicroRNA Disease Database), and dbDEMC (database of Differentially Expressed MiRNAs in human Cancers), which are invaluable resources for exploring miRNA functional interactions [15,16,17,18]. Some methods infer the miRNA interactions based on the knowledge bases. DDRM (defining disease-related modules) [9] measures the miRNA functional interactions by considering the co-regulating target subset and the non-common target set of miRNAs to construct the network, and identifies the key modules by integrating the miRNA expression data. Moreover, under the assumption that miRNAs with similar functions tend to be associated with similar diseases, some studies used Gaussian interaction profile kernel similarity to evaluate miRNA functional interactions based on miRNA-disease associations [19,20,21]. The knowledge about miRNA-disease associations can provide direct evidence that miRNAs are involved in the onset and progression of diseases [22].

Despite these advancements, identifying potential miRNA biomarkers from the network constructed only by using miRNA expression data or knowledge from databases still has limitations. The limitation of the network constructed purely based on knowledge is that public knowledge bases are seldom disease-specific and may not accurately reflect miRNA interactions for a specific disease. Furthermore, they may have some false negatives due to the incompleteness of knowledge bases [23]. In contrast, the network constructed based on the experimental data may have some false positives due to the sample size, the sample coverage, and the background noise [23]. Given this, it is beneficial to obtain a more accurate and stable network by the joint use of knowledge from databases and miRNA expression data. One easy method is to simply average the miRNA interactions from two sources [22], which mixes the local structure of the network derived from each source [24]. Now, it remains challenging to generate a robust network by appropriately leveraging knowledge from databases and miRNA expression data so that it can better reflect miRNA interactions in disease and help to define more reliable and biologically meaningful miRNA biomarkers.

With the development of deep learning, graph neural networks (GNNs) have emerged as an effective tool for analyzing graph data, and graph autoencoders (GAEs) are an important part of GNNs [25]. GAEs are unsupervised learning frameworks that are generally composed of an encoder and a decoder. The encoder maps node attributes and graph structure into lower-dimensional latent representations, and the decoder reconstructs the graph information from the latent information. The encoder-decoder structure of GAEs endows them with superior node representation ability under unsupervised learning, which has been successfully used in some bioinformation applications, such as drug-disease association prediction and single-cell multi-omics data clustering [26,27,28].

This study proposes a disease-related miRNA biomarker identification method based on the knowledge-enhanced bio-network (named BIM-Ken), jointly utilizing miRNA expression data and knowledge from public databases. Figure S1 (see Supplementary Information) shows the overview of BIM-Ken. BIM-Ken utilizes GAE to optimize the experimental data-based network to acquire the knowledge-enhanced bio-network, owing to the superior capability of GAE in handling graph data and preserving intrinsically important information [29]. Specifically, BIM-Ken first generates a miRNA cooperation network based on the expression data and enriches the node attributes through the knowledge about miRNA-disease associations. Then, the cooperation network is fed into the GAE, and the reconstruction loss, the functional consistency constraint, and the difference prompt constraint are introduced to learn the synergy relationship, functional information, and disease-specific relationship between miRNAs. The learned representations are used to infer the knowledge-enhanced network, i.e., miRNA interaction network. Finally, BIM-Ken identifies the modules from the miRNA interaction network by a greedy searching strategy and selects the important modules as the disease-related miRNA biomarkers. Experiments on the public datasets and the application of the miRNA expression dataset associated with renal cell carcinoma showed the validity of BIM-Ken.

## 2. Materials and Methods

### 2.1. Data

In this work, nine miRNA expression datasets downloaded from the Gene Expression Omnibus (GEO) database (https://www.ncbi.nlm.nih.gov/gds/, accessed on 16 June 2024) [30] were adopted to evaluate the effectiveness of BIM-Ken. Table 1 provides the detailed descriptions of the nine datasets. The datasets are related to multiple diseases, such as prostate cancer, breast cancer, and gastric cancer. For each dataset, if one probe corresponds to multiple miRNA names, it was removed. When multiple probes are mapped to the same miRNA name, only the one with the highest average expression level across all samples was retained. Moreover, all human mature miRNA names were converted to their corresponding miRNA accession numbers (MIMAT IDs) based on the miRBase database (miRBase version 22.0) [31] using the miRNAmeConverter package [32], and any miRNAs that could not be converted to MIMAT IDs were removed.

The knowledge about miRNA-disease associations was extracted from two widely used knowledge bases dbDEMC [17] and miRCancer [18]. In dbDEMC, the focus is on the differentially expressed miRNAs in various diseases. The miRCancer database utilizes text mining techniques to extract miRNA and disease associations from the published literature.

### 2.2. BIM-Ken Method

**Figure 1:**
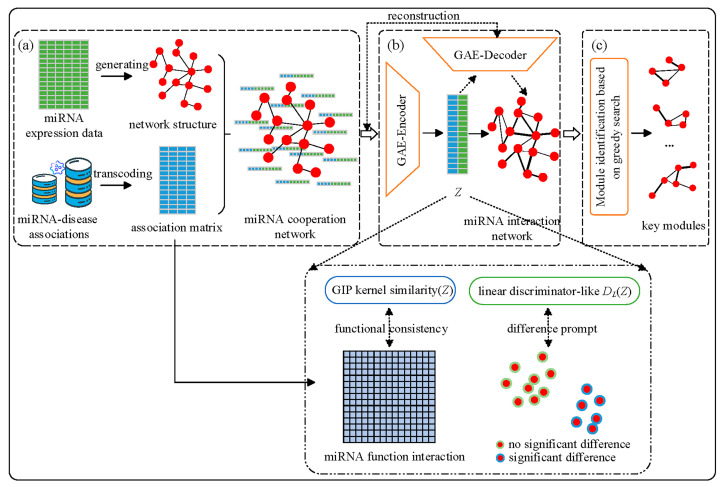
Workflow of BIM-Ken (**a**) miRNA cooperation network generation, (**b**) miRNA cooperation network enhancement, (**c**) key miRNA module identification.

The proposed BIM-Ken consists of three steps: miRNA cooperation network generation, miRNA cooperation network enhancement, and key miRNA module identification. Figure 1 shows the workflow of BIM-Ken.

#### 2.2.1. MiRNA Cooperation Network Generation

Let *S* = {*s*_1_, *s*_2_, …, *s_n_*} be the training sample set with *n* samples, *F* = {*f*_1_, *f*_2_, …, *f_m_*} be the feature (miRNA) set, and *m* be the number of features. Table S1 (see Supplementary Information) lists a summary of key mathematical symbols and notations used in the study.

The relationships of miRNA *f_i_* (1 ≤ *i* ≤ *m*) and the diseases are represented as a binary vector $d(f_{i})=(d_{1}^{f_{i}},d_{2}^{f_{i}},\ldots ,d_{p}^{f_{i}})$, in which *p* is the number of the diseases [19]. Specifically, if miRNA *f_i_* is associated with the disease *d_t_* (*t* = 1, 2, …, *p*), then $d_{t}^{f_{i}}$ is 1, otherwise, $d_{t}^{f_{i}}$ is 0.

For two features *f_i_* and *f_j_* (1 ≤ *i* ≠ *j* ≤ *m*), SVM (Support Vector Machine) with linear kernel can find an optimal hyper-plane *α_ij_ f_i_* + *β_ij_ f_j_* + *γ_ij_* = 0 that maximizes the decision boundary, enhancing model generalization and minimizing classification errors [33]. This hyper-plane illustrates the ability of the cooperation of *f_i_* and *f_j_* to separate different sample groups. Hence, BIM-Ken defines the artificial combinatorial feature *f_com_*(*f_i_*, *f_j_*) = *α_ij_ f_i_* + *β_ij_ f_j_* + *γ_ij_* to represent the linear combinatorial relationship between miRNA *f_i_* and miRNA *f_j_*. It is believed that the linear relationship is simpler, and it is easier to obtain a biomedical explanation than the non-linear relationship. Moreover, the differences in the expression levels of *f_com_*(*f_i_*, *f_j_*) between the distinct sample groups reflect the alteration in the cooperative regulation of miRNA *f_i_* and miRNA *f_j_*. Here, the *t*-test is introduced to examine the significant change of *f_com_*(*f_i_*, *f_j_*) in different sample groups. The cooperative regulation of *f_i_* and *f_j_* is strong if *f_com_*(*f_i_*, *f_j_*) changes significantly in different sample groups (i.e., *p*-value(*f_com_*(*f_i_*, *f_j_*)) < 0.05). Thus, the miRNA cooperation network is defined based on significant differences in the artificial combinatorial features between different sample groups.

Let *G_co_* = (*V_co_*, *E_co_*) be the undirected cooperation network, then the node set is the input feature set (i.e., *V_co_* = *F*), there is an edge between *f_i_* and *f_j_* if *p*-value (*f_com_*(*f_i_*, *f_j_*)) < 0.05, i.e., *E_co_* = {(*f_i_*, *f_j_*)|*f_i_*, *f_j_* ∈*F*, 1 ≤ *i* ≠ *j* ≤ *m*, *p*-value(*f_com_*(*f_i_*, *f_j_*)) < 0.05}. Also, the attributes of each node are denoted as *x_co_*(*f_i_*) = *ѱ*(*d*(*f_i_*), *s*(*f_i_*)), where $s(f_{i})=(s_{1}^{f_{i}},s_{2}^{f_{i}},\ldots ,s_{n}^{f_{i}})$ is the expression values of miRNA *f_i_* on all samples, and the *ѱ*() denotes the concatenation operation. The attributes of all the nodes in *G_co_* form an *m* × (*p + n*) node attribute matrix *X_co_*. In this way, a cooperation network is established, the network topology is induced by the miRNA expression data, and the attributes of the network node contain the values of the corresponding miRNAs on the samples and their knowledge information associated with diseases.

#### 2.2.2. MiRNA Cooperation Network Enhancement

The cooperation network *G_co_* is constructed based on the miRNA expression data, and the node information is enriched by prior knowledge. The network structure is determined based on the miRNA data, which is affected by factors such as sample size, sampling coverage, and background noise of the data. Small sample size, limited sampling coverage, and noise may lead to false positives. The knowledge about miRNA-disease associations offers direct evidence that miRNAs participate in the occurrence and development of certain diseases, and it is invaluable for exploring miRNA functional interactions. Hence, to optimize the network and mitigate false positives, GAE is used to fuse experimental data and prior knowledge to enhance the latent representations of miRNAs and then optimize the network.

Let *A_co_* be the adjacency matrix of *G_co_*. BIM-Ken uses the encoder of GAE to extract the latent representations of the nodes in *G_co_*. The encoder is defined as follows [34]:

$$Z={\tilde{D}}^{-\frac{1}{2}}\tilde{A}{\tilde{D}}^{-\frac{1}{2}}\sigma ({\tilde{D}}^{-\frac{1}{2}}\tilde{A}{\tilde{D}}^{-\frac{1}{2}}XW_{0})W_{1}$$

where $\tilde{A}=A_{co}+I_{m}$ is the adjacency matrix *A_co_* with added self-connections, *I_m_* is the identity matrix, $\tilde{D}$ is the diagonal degree matrix of $\tilde{A}$, *X* = *X_co_*, *W*_0_ and *W*_1_ are the trainable weight matrices, and σ() is the exponential linear unit (ELU) activation function [35]. Formula (1) enables the representations *Z* of nodes to effectively integrate the information of node relationships and node attributes in the cooperative network graph *G_co_*, which fully exploits the knowledge about miRNA-disease associations and miRNA expression data jointly.

The latent representations *Z* should encapsulate the essential information of the input data, thereby facilitating the reconstruction of the network. Based on a general biological assumption that the miRNAs with similar functions tend to be associated with similar diseases, BIM-Ken considers the miRNA functional interactions based on the miRNA-disease associations knowledge and preserves the consistency between the similarity of learned node representations and the miRNA functional interactions [19,20,21]. Also, pathogenic alterations typically result in some differentially expressed molecules [36]. Capturing the distinction between differentially and non-differentially expressed molecules helps to learn finer representations and facilitates downstream network analysis tasks, including potential disease biomarker identification. With regard to this, the loss function consists of three parts: the reconstruction loss, the functional consistency constraint, and the difference prompt constraint.

(1) The reconstruction loss

Refer to GAE, the inner product decoder reconstructs the input from the latent representations *Z*, and the reconstructed adjacency matrix *A_rec_* is formulated by:

*A_rec_ = sigmoid*(*ZZ^T^*)

BIM-Ken uses the mean squared error (MSE) as the reconstruction loss between the input adjacency matrix *A_co_* and the reconstructed adjacency matrix *A_rec_* [37]:

*L_rec_ = MSE*(*A_co_*, *A_rec_*)

Based on the above reconstruction process, the extracted representations can inherit more information from the cooperation network, which helps retain the cooperative regulation of miRNAs from the sample data.

(2) Functional consistency constraint

To capture miRNA functional interactions, Gaussian interaction profile (GIP) kernel similarity is adopted to calculate the similarity of miRNAs between *d*(*f_i_*) of miRNA *f_i_* and *d*(*f_j_*) of miRNA *f_j_* (1 ≤ *i* ≠ *j* ≤ *m*), and obtain the GIP kernel similarity matrix *GS* ∈${\mathbb{R}}^{m\times m}$:

$$GS_{ij}=\exp(-r_{m}∥d(f_{i})-d(f_{j}){∥}^{2})$$

$$r_{m}=r_{m}^{\prime }/(\frac{1}{m}\sum_{k=1}^{m}∥d(f_{k}){∥}^{2})$$

where $r_{m}^{\prime }$ is set to 1 according to the study [19], and exp() is the exponential function. Analogously, the GIP kernel similarity is also used to measure the pairwise similarity between the representation *Z_i_* and the representation *Z_j_* to obtain *GS’*∈${\mathbb{R}}^{m\times m}$ (*Z_i_* represents the learned representations of node *f_i_* (1 ≤ *i* ≠ *j* ≤ *m*)). The functional consistency constraint *L_fc_* is introduced to preserve the consistency between *GS* and the *GS’* [38]:

$$L_{fc}=∥GS-GS^{\prime }{∥}_{F}^{2}$$

(3) Difference prompt constraint

Inspired by prompt learning in natural language processing, the difference prompt constraint was designed to enable learned node representations to behave discrepantly between differentially expressed and non-differentially expressed miRNAs. Assume a pseudo-label *Y^P^* for each node. If the *p*-value from the *t*-test for feature *f_i_* is less than 0.05, then *Y^p^*(*f_i_*) = *y*_0_; otherwise, *Y^p^*(*f_i_*) = *y*_1_. A linear discriminator-like *D_L_*() is trained to partition nodes based on the latent representations *Z*. The difference prompt constraint *L_mp_* is defined as follows [39,40]:

$$L_{mp}=\sum_{f_{i}\in F}\frac{-1}{\left|B(f_{i})\right|}\sum_{b\in B(f_{i})}\log\frac{\exp(D_{L}(Z_{i})•D_{L}(Z_{b})/\tau )}{\sum_{a\in A(f_{i})}\exp(D_{L}(Z_{i})•D_{L}(Z_{a})/\tau )}$$

where *A*(*f_i_*) = *F*\{ *f_i_* } is the subset of all features excluding *f_i_*, *B*(*f_i_*) = {*b*∈ *A*(*f_i_*): *Y^P^*(*b*) = *Y^P^*(*f_i_*)} is the subset of the features with the same pseudo label as *f_i_*, the symbol *•* denotes the inner (dot) product operation, *τ* is a scalar temperature parameter, |*B*(*f_i_*)| denotes its cardinality.

The reconstruction loss, the functional consistency constraint, and the difference prompt constraint constitute the overall objective function of BIM-Ken, which is defined as follows:

*L_total_* = *L_rec_* + *λ_1_L_fc_* + *λ_2_L_mp_*

where *λ*_1_ and *λ*_2_ are the balance factors.

The final latent representations of the nodes learn the synergy relationship, functional information, and disease-specific relationship between miRNAs. Based on the enhanced latent representations of the nodes, BIM-Ken builds the enhanced interaction network *G_Net_*.

#### 2.2.3. Key miRNA Module Identification

To identify the miRNA information module in *G_Net_*, a greedy searching strategy is provided. Thereinto, the performance of a module is evaluated by the area under the curve (AUC) value, which is computed based on the features (nodes) in the module.

(1) Initially, the current module contains the most important node in *G_Net_*. The candidate node refers to the node adjacent to the edge having the highest edge weight among all the edges connected to the current module in *G_Net_* (in instances where multiple candidate nodes exist, the one that demonstrates the largest performance when it is added to the current module is selected). In each extension, the candidate node is examined. If the candidate node can improve the performance of the current module, it is added to the module; otherwise, it will be added to the current module with a probability *Prob_t_ =* 1 − exp((*AUC_cmn_* − 1)/*cmn*), where *cmn* represents the number of nodes including both the current candidate node and nodes in the current module, and *AUC_cmn_* represents the AUC value of the current module and the candidate node. The *Prob_t_* is considered to avoid the local optimal in the searching procedure. This extension stops when no candidate node can be added to the current module.

When a module searching procedure terminates, the nodes in the identified module are removed from *G_Net_*.

(2) The procedure (1) is repeated until the edge set in *G_Net_* is empty or the preset maximum number of modules *Maxmodulenum* is reached.

Since the edge weight reflects the strength of the interaction between two adjacent nodes, BIM-Ken evaluates the node importance by the sum of the weights of the edges adjacent to the node, which represents the influence of the node in the network.

Among the detected modules, the *k* > 0 ones with the highest AUC values are defined as the key modules. For the *k* modules, the SVM classifiers with the linear kernel are trained, and the final prediction is decided by the majority voting of the *k* base SVM classifiers.

## 3. Experimental Settings

To evaluate the performance of BIM-Ken, data-driven analysis methods and the hybrid method, which combines miRNA expression data and prior knowledge, were chosen as the baselines, including the popular machine learning technique support vector machine-recursive feature elimination (SVM-RFE) [41], network analysis methods INtegrated DiffErential Expression and Differential network analysis (INDEED) [11], GRAph Convolutional nEtwork feature Selector (GRACES) [42], Graph Convolutional Network-based approach for Clustering and Classification (GCNCC) [43], NetRank [44], and the defining disease-related modules (DDRM) method, as well as the common statistical technique *t*-test.

SVM-RFE and *t*-test are the most commonly used and fundamental methods for omics data analysis. GRACES is a graph neural network-based method that exploits relationships between samples and uses various overfitting-reducing techniques to find an optimal feature subset. INDEED builds the differential correlation network based on the partial correlation and integrates molecule differential expression and differential network analysis for biomarker discovery. GCNCC learns deep network representations by integrating gene expression data and the existing network, then applies the geometric affinity propagation (Geometric-AP) to cluster network nodes and screens the cluster with discriminatory ability. This study used GCNCC to analyze miRNA expression data and constructed the network using PCC. NetRank uses the weighted gene correlation network analysis (WGCNA) method to construct the molecular network, and prioritizes and selects disease-related biomarkers by a random surfer mode. DDRM identifies the module biomarkers by combining the knowledge databases and miRNA expression data. It constructs the weighted miRNA synergistic network by the co-regulating target subset and the non-common target set and identifies miRNA synergistic modules. Then, DDRM maps the miRNAs in the miRNA expression data to the identified network modules and defines the module biomarkers based on the classification performance.

In the experiment, INDEED and *t*-test ranked features according to their respective weights, and the top *r* (*r* ∈ [1, min{100, |*F*|}], in which *F* is the set of remaining features after preprocessing in INDEED, and *F* refers to the set of features with *p*-values less than 0.05 in *t*-test) features with the highest classification performance using the sequential forward searching were selected. The number of selected modules of DDRM was set to 7. For BIM-Ken, the parameters *λ*_1_, *λ*_2_, *Maxmodulenum*, learning rate, the number of epochs, the temperature parameter *τ*, and the module number *k* were set to 0.1, 1 × 10^−7^, 10, 0.01, 200, 0.1, and 7, respectively (see Supplementary Information). Moreover, a negative sampling strategy was used in the model training process. SVM with the linear kernel was used as a classifier for all the methods, except GCNCC, which uses the logistic regression [45] according to [43].

The implementation of BIM-Ken was written in Python 3.8. Also, ten-fold cross-validation was run 10 times to obtain the average performance of each method.

## 4. Results and Discussion

### 4.1. Performance Comparison

In this section, BIM-Ken is compared with SVM-RFE, INDEED, GRACES, *t*-test, GCNCC, NetRank, and DDRM methods in classification accuracy rate, sensitivity, and specificity. The results are shown in Table 2, Table 3 and Table 4, where “Ave” represents the average classification performance of each method over the nine public datasets, and “W/T/L” (win/tie/lose) represents the number of datasets on which BIM-Ken achieved higher or equal or lower classification performance than the compared method.

Table 2 shows that BIM-Ken outperformed SVM-RFE, INDEED, GRACES, *t*-test, GCNCC, NetRank, and DDRM in most cases in terms of classification accuracy rate. BIM-Ken outperformed SVM-RFE, INDEED, GRACES, *t*-test, GCNCC, NetRank, and DDRM in classification accuracy rate for nine, nine, eight, nine, nine, nine, and nine of the nine datasets, and it significantly outperformed SVM-RFE, INDEED, GRACES, *t*-test, GCNCC, NetRank, and DDRM for nine, nine, seven, six, nine, five, and seven of the nine datasets.

Moreover, the sensitivity and specificity were also examined to further evaluate the performance of each method. The sensitivity calculates the proportion of true positives. The specificity measures the proportion of true negatives. Table 3 and Table 4 show that the sensitivity and specificity of BIM-Ken were higher than those of SVM-RFE, INDEED, GRACES, *t*-test, GCNCC, NetRank, and DDRM in most cases, and BIM-Ken obtained the highest average sensitivity and the highest average specificity over all the datasets.

Overall, BIM-Ken has advantages in defining powerful disease biomarkers and predicting sample labels. The comparison of BIM-Ken and data-driven analysis methods (e.g., SVM-RFE, INDEED, and GRACES) demonstrates that combining the miRNA expression data with prior knowledge can provide comprehensive insights to identify critical information reflecting the physiological and pathological changes. In addition, the comparison of BIM-Ken and the hybrid method (i.e., DDRM) shows that BIM-Ken is an effective method to appropriately leverage knowledge from databases and miRNA expression data, and the acquired miRNA interaction network can better reflect the miRNA interaction in disease and subsequently help to find more reliable miRNA biomarkers.

### 4.2. Ablation Study

BIM-Ken employs GAE to optimize the experimental data-based miRNA cooperation network. A functional consistency constraint is introduced into representation learning to preserve the consistency between the similarity of learned node representations and the miRNA functional interactions, and the difference prompt constraint is designed to enable learned node representations to capture the distinction between differentially expressed and non-differentially expressed miRNAs.

To validate the effectiveness of functional consistency based on the knowledge, difference prompt, and optimizing the cooperation network by GAE, we compared BIM-Ken with its three variations, (1) BIM-Ken-w/o-KI, indicating that the node attributes in the miRNA cooperation network do not contain knowledge information and the functional consistency constraint is removed, (2) BIM-Ken-w/o-DP, indicating that the difference prompt constraint is removed, and (3) BIM-Ken-w/o-GAE, indicating that optimizing miRNA cooperation network by GAE is removed (i.e., the key module identification and selection are performed on the miRNA cooperation network based on the experimental data).

**Figure 2:**
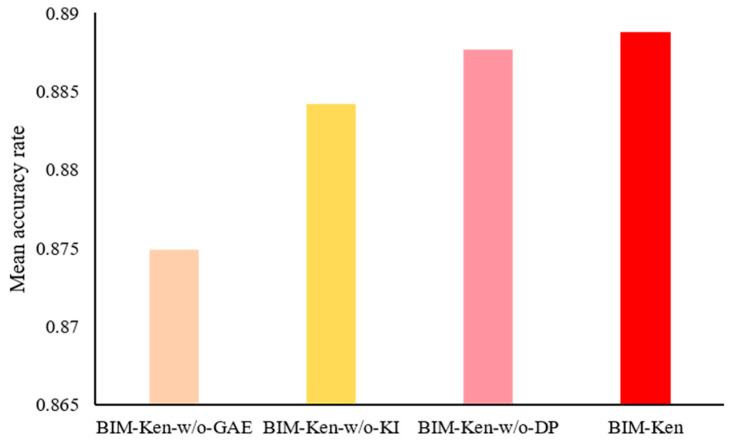
Comparison of BIM-Ken with BIM-Ken-w/o-GAE, BIM-Ken-w/o-KI, and BIM-Ken-w/o-DP in average classification accuracy rate.

Figure 2 shows the comparison results in the average classification accuracy rate over all datasets. It can be seen that BIM-Ken outperformed BIM-Ken-w/o-KI and BIM-Ken-w/o-DP, demonstrating the rationality and effectiveness of functional consistency based on the knowledge and difference prompt. Moreover, BIM-Ken also outperformed BIM-Ken-w/o-GAE, suggesting that optimizing the experimental data-based miRNA cooperation network by GAE can enhance the cooperation network. The optimized network can better reflect miRNA interactions in disease, which is conducive to the identification of more reliable and biologically meaningful miRNA biomarkers.

### 4.3. Module Biomarker Detected by BIM-Ken for the Renal Cell Carcinoma

Renal cell carcinoma (RCC) is a prevalent malignant tumor of the urinary system, and clear cell renal cell carcinoma (ccRCC) is the most common histologic subtype of RCC [46]. BIM-Ken is applied to analyze the miRNA expression data of ccRCC to identify the miRNA biomarkers and facilitate the diagnosis study of renal cell carcinoma. The discovery set about ccRCC from GEO, accession number GSE116251 [47], includes 18 pairs of tumor and adjacent normal-tissue samples. An independent validation set was retrieved from the TCGA (The Cancer Genome Atlas) (https://www.cancer.gov/tcga, accessed on 10 January 2024), i.e., Kidney Clear Cell Carcinoma (KIRC), which was downloaded from UCSC Xena (http://xena.ucsc.edu, accessed on 10 January 2024) [48].

**Figure 3:**
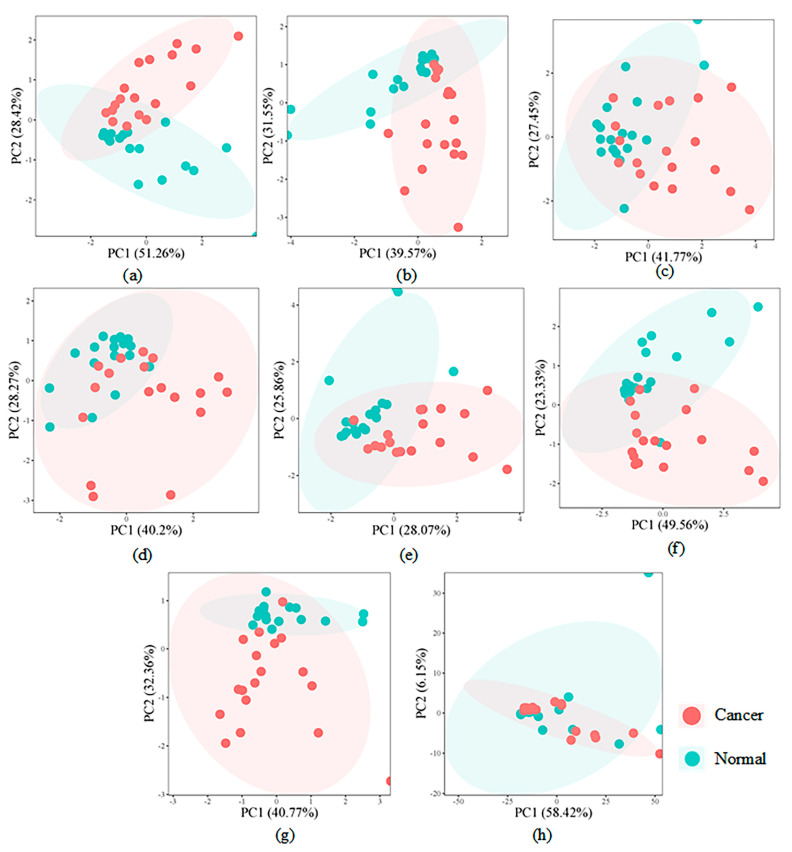
PCA score scatter plots (**a**) M_1 module, (**b**) M_2 module, (**c**) M_3 module, (**d**) M_4 module, (**e**) M_5 module, (**f**) M_6 module, (**g**) M_7 module, (**h**) all input features.

For the discovery set (GSE116251), the detailed descriptions of key modules identified by BIM-Ken are shown in Table S2 (see Supplementary Information). To validate the discriminative ability of the defined modules, Figure 3 shows the score scatter plots of principal component analysis (PCA) based on all input features and the defined modules. As shown in Figure 3a–g, the PCA plots of most defined modules demonstrate a clear separation trend between ccRCC samples and healthy control samples. In contrast, PCA plots derived from all input features (see Figure 3h) show that ccRCC and healthy control samples are mixed.

The independent validation dataset KIRC was mapped to these key modules, SVM was used as the classifier, and the classification performance is given in Table 5. It can be seen that these modules achieved good classification performance in distinguishing between cancer samples and non-cancer samples and showed great potentiality as biomarkers for renal cell carcinoma.

We further investigated the relationship between the defined modules and renal cell carcinoma, under the assumption that if the module contains the known cancer-related miRNAs, the module is associated with the corresponding cancer [9]. Table S2 (see Supplementary Information) lists key modules identified by BIM-Ken, where the symbol “#” represents the module related to renal cell carcinoma. This was conducted based on the HMDD [16]. It is evident that all the defined modules are related to renal cell carcinoma, reflecting that these modules play an important role in the occurrence and development of renal cell carcinoma.

To explore the function of the defined modules, KEGG (Kyoto Encyclopedia of Genes and Genomes) pathway analysis was conducted for the target genes of the identified key modules [49,50]. Table 6 shows the enriched representative pathways for the target genes of each identified key module, which were conducted using the DAVID tools [51]. Most of them are significantly enriched in the renal cell carcinoma (hsa05211) pathway. Additionally, some modules are significantly enriched in several critical signal transduction pathways, such as the MAPK signaling pathway (hsa04010) and TGF-beta signaling pathway (hsa04350), which play pivotal roles in renal cell carcinoma [52]. These indicate that the target genes of the identified key modules are closely related to renal cell carcinoma.

**Figure 4:**
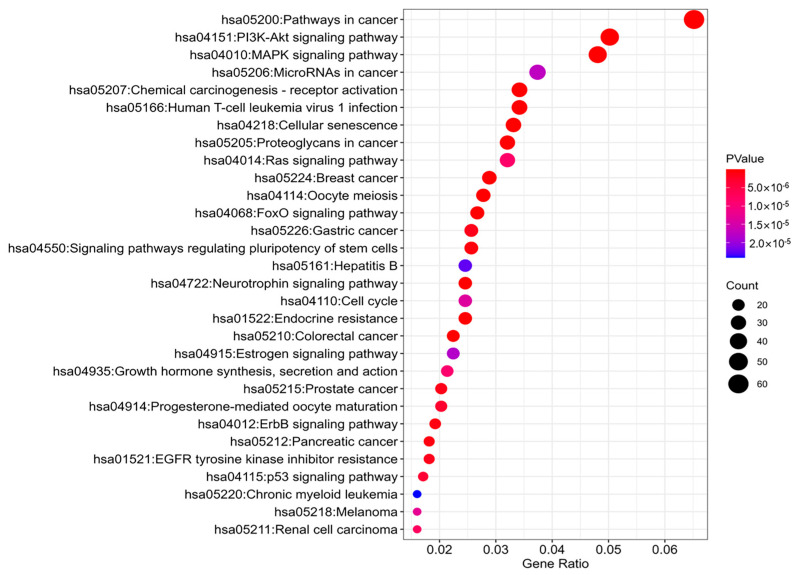
Top 30 enriched KEGG pathways for target genes of M6-associated miRNAs.

Moreover, Module M_6 is significantly enriched in the renal cell carcinoma (hsa05211) pathway with a *p*-value of 7.51 × 10^−6^. Figure 4 shows the top 30 KEGG pathways enriched for target genes of M_6-associated miRNAs. These pathways are related to renal cell carcinoma. Typically, the PI3K-Akt signaling pathway (hsa04151) consists of multiple bifurcating and converging kinase cascades and is highly activated in the RCC and modestly mutated, which is a promising drug target, and the PI3K pathway inhibitors of the rapalog family are approved for use in RCC [53]. Hsa-miR-15a-5p, hsa-miR-222-3p, and hsa-miR-509-3p in M_6 are closely associated with renal cell carcinoma [54,55,56]. Hsa-miR-15a-5p is involved in cellular proliferation, migration, invasion, and apoptosis in renal cancer cell lines, which indicates that hsa-miR-15a-5p acts as an oncogene in RCC [54]. Hsa-miR-222-3p functions to promote renal cell carcinogenesis. From a molecular perspective, high expression of hsa-miR-222-3p is associated with increased metastatic potential and decreased apoptosis in vitro. Also, hsa-miR-222-3p targets TIMP2 (a tumor suppressor) and ERK1/2 to deliver its oncogenic functions [55]. Hsa-miR-222-3p may be used as a biomarker and therapeutic target for RCC. Furthermore, the overexpression of hsa-miR-509-3p suppresses the mRNA and protein expression levels of MAP3K8, and the knockdown of MAP3K9 inhibits the migration and proliferation of RCC cells, suggesting that the hsa-miR-509-3p RCC suppressor serves as a crucial regulator of the MAP3K8 oncogene, potentially offering therapeutic implications for the treatment of RCC [56].

In summary, BIM-Ken has demonstrated effectiveness in identifying potential miRNA biomarkers for renal cell carcinoma, and further research on the role of these miRNAs in the development of renal cell carcinoma is essential.

## 5. Conclusions

In this study, we propose a disease-related miRNA biomarker identification method based on the knowledge-enhanced bio-network (BIM-Ken), jointly utilizing miRNA expression data and knowledge from public databases. BIM-Ken constructs the miRNA cooperation network based on miRNA expression data and enriches the node attributions through prior knowledge. Benefiting from the excellent ability of GAE to handle graph data and retain intrinsic information, GAE is adopted to optimize the miRNA cooperation network and generate a more robust network. In this process, functional consistency is introduced to preserve the consistency between the similarity of learned node representations and the miRNA functional interactions, and the difference prompt is designed to enable learned node representations to discriminate between differentially expressed and non-differentially expressed miRNAs. BIM-Ken develops a greedy searching strategy to identify the modules from the network as the disease-related biomarkers. The comparisons of BIM-Ken with the data-driven analysis methods and the hybrid method, which combines the miRNA expression data and prior knowledge on the nine public datasets, showed the effectiveness of BIM-Ken. Furthermore, the application of BIM-Ken to the renal cell carcinoma data illustrated that the biomarkers identified by BIM-Ken could better distinguish between cancer and non-cancer samples and had biological significance. In brief, BIM-Ken provides a new way to explore the miRNA interaction network by combining knowledge from the public database and miRNA expression data, which can better measure the miRNA interaction in disease and help to identify more reliable and biologically meaningful miRNA biomarkers. Especially, BIM-Ken relied on miRNA-disease associations from public databases to enhance the network. Although the databases are widely used and provide carefully curated resources, they are inherently incomplete. It is expected that continuous updates and enrichments to the databases will improve the accuracy of the constructed network.

## Figures and Tables

**Table 1:** Details of the nine miRNA expression datasets.

Datasets	Disease Types	Features	Samples	Classes
GSE34496	Head and neck squamous cell carcinoma	812	69	2
GSE36802	Prostate cancer	812	42	2
GSE41922	Breast cancer	264	54	2
GSE67139	Hepatocellular carcinoma	812	115	2
GSE76260	Prostate cancer	787	64	2
GSE78775	Gastric cancer	818	56	2
GSE116251	Renal cell carcinoma	769	36	2
GSE142699	Acute myeloid leukemia	769	48	2
GSE158284	Glioblastoma	214	41	2

**Table 2:** Comparison in classification accuracy rate (Mean ± S.D.%).

Datasets	BIM-Ken	SVM-RFE	INDEED	GRACES	*t*-Test	GCNCC	NetRank	DDRM
GSE34496	**94.50 ± 1.90**	87.36 ± 3.70 *	91.62 ± 2.70 *	91.38 ± 2.02 *	93.64 ± 2.61	85.43 ± 4.46 *	91.86 ± 1.84 *	89.98 ± 1.86 *
GSE36802	**93.10 ± 3.89**	82.50 ± 5.90 *	87.40 ± 1.82 *	91.95 ± 3.83	87.55 ± 3.60 *	84.85 ± 4.96 *	91.95 ± 2.66	89.50 ± 3.79
GSE41922	90.57 ± 1.46	85.70 ± 2.28 *	84.73 ± 3.31 *	**91.43 ± 2.89**	86.07 ± 3.15 *	87.00 ± 4.11 *	86.77 ± 2.01 *	85.83 ± 3.56 *
GSE67139	**87.17 ± 1.84**	77.35 ± 1.73 *	82.92 ± 1.83 *	81.48 ± 3.09 *	84.14 ± 1.72 *	83.52 ± 2.67 *	85.18 ± 2.94	81.06 ± 2.04 *
GSE76260	**79.38 ± 2.92**	72.57 ± 5.67 *	74.88 ± 3.76 *	71.67 ± 2.59 *	73.10 ± 3.09 *	71.17 ± 3.62 *	74.74 ± 4.24 *	72.07 ± 3.24 *
GSE78775	**80.23 ± 2.64**	74.37 ± 5.57 *	74.33 ± 4.85 *	68.80 ± 6.08 *	77.50 ± 3.21	58.97 ± 3.62 *	79.87 ± 3.42	69.10 ± 4.59 *
GSE116251	**85.33 ± 4.40**	78.33 ± 3.97 *	80.42 ± 2.95 *	73.58 ± 4.55 *	83.25 ± 4.15	64.00 ± 11.55 *	82.83 ± 4.07	79.25 ± 3.63 *
GSE142699	**97.40 ± 0.91**	91.80 ± 2.54 *	91.75 ± 3.08 *	94.80 ± 1.44 *	93.95 ± 3.18 *	94.60 ± 3.55 *	94.50 ± 1.97 *	95.55 ± 3.02
GSE158284	**92.20 ± 3.12**	83.85 ± 1.86 *	86.05 ± 2.58 *	85.95 ± 3.82 *	84.70 ± 3.05 *	85.30 ± 4.46 *	82.85 ± 3.35 *	87.95 ± 2.90 *
Ave	**88.88**	81.54	83.79	83.45	84.88	79.43	85.62	83.37
W/T/L		9/0/0	9/0/0	8/0/1	9/0/0	9/0/0	9/0/0	9/0/0

Bold: the highest accuracy rate for the corresponding dataset. *: BIM-Ken statistically significant (at 0.05 level) wins or loses the corresponding method.

**Table 3:** Comparison in sensitivity (Mean ± S.D.%).

Datasets	BIM-Ken	SVM-RFE	INDEED	GRACES	*t*-Test	GCNCC	NetRank	DDRM
GSE34496	**94.60 ± 1.79**	89.05 ± 4.56 *	91.45 ± 2.31 *	92.05 ± 2.93 *	93.60 ± 1.87	92.40 ± 3.92	91.40 ± 2.65 *	91.55 ± 2.95 *
GSE36802	89.17 ± 5.57	76.00 ± 9.00 *	86.17 ± 3.93	**91.33 ± 5.32**	84.83 ± 3.55	89.17 ± 4.25	90.33 ± 3.91	88.17 ± 5.47
GSE41922	90.67 ± 2.22	85.25 ± 3.38 *	86.92 ± 5.11	92.17 ± 3.50	85.08 ± 4.88 *	**92.75 ± 4.36**	87.08 ± 3.65 *	86.75 ± 2.87 *
GSE67139	84.93 ± 2.14	73.67 ± 1.85 *	80.57 ± 2.72 *	78.67 ± 3.09 *	81.00 ± 2.01 *	**86.03 ± 2.83**	85.20 ± 3.92	80.00 ± 3.23 *
GSE76260	**76.00 ± 4.81**	70.25 ± 6.57 *	74.42 ± 7.73	73.33 ± 6.21	72.83 ± 6.14	73.50 ± 4.76	72.83 ± 7.83	70.42 ± 6.10 *
GSE78775	79.17 ± 3.54	75.00 ± 6.48	75.67 ± 4.73	67.00 ± 9.78 *	77.00 ± 4.43	62.00 ± 4.89 *	**82.67 ± 6.68**	67.33 ± 5.89 *
GSE116251	**79.50 ± 7.62**	73.50 ± 7.84	76.50 ± 10.01	73.50 ± 5.80	78.00 ± 8.56	69.00 ± 9.94 *	79.00 ± 6.99	**79.50 ± 5.99**
GSE142699	**94.50 ± 2.09**	90.00 ± 2.36 *	92.67 ± 4.39	92.33 ± 2.96	93.00 ± 4.29	93.00 ± 4.29	91.00 ± 3.06 *	92.33 ± 5.73
GSE158284	**97.17 ± 3.34**	87.00 ± 2.05 *	90.17 ± 3.55 *	89.00 ± 5.73 *	88.83 ± 4.01 *	90.33 ± 6.18 *	85.33 ± 2.46 *	89.83 ± 2.77 *
Ave	**87.30**	79.97	83.84	83.26	83.80	83.13	84.98	82.88
W/T/L		9/0/0	9/0/0	7/0/2	9/0/0	6/1/2	6/0/3	8/1/0

Bold: the highest sensitivity for the corresponding dataset. *: BIM-Ken statistically significant (at 0.05 level) wins or loses the corresponding method.

**Table 4:** Comparison in specificity (Mean ± S.D.%).

Datasets	BIM-Ken	SVM-RFE	INDEED	GRACES	*t*-Test	GCNCC	NetRank	DDRM
GSE34496	**94.17 ± 2.86**	84.50 ± 6.67 *	92.33 ± 5.10	89.67 ± 4.07 *	93.67 ± 3.83	73.83 ± 6.76 *	92.67 ± 2.96	86.67 ± 3.69 *
GSE36802	**96.83 ± 4.68**	88.50 ± 8.29 *	89.00 ± 5.34 *	92.50 ± 3.54 *	90.33 ± 4.96 *	81.00 ± 6.54 *	93.67 ± 4.83	90.83 ± 4.39 *
GSE41922	**90.83 ± 3.36**	86.17 ± 4.01 *	82.17 ± 4.72 *	90.50 ± 5.78	87.50 ± 4.32	79.67 ± 6.47 *	87.00 ± 3.31 *	84.00 ± 6.05 *
GSE67139	**89.50 ± 2.17**	81.03 ± 4.57 *	85.40 ± 2.11 *	84.67 ± 4.22 *	87.37 ± 2.49	81.43 ± 3.45 *	85.30 ± 3.63 *	81.93 ± 2.32 *
GSE76260	**82.58 ± 3.59**	74.83 ± 6.26 *	75.08 ± 5.59 *	69.75 ± 2.83 *	73.42 ± 3.25 *	69.17 ± 3.26 *	76.08 ± 6.16 *	73.92 ± 4.16 *
GSE78775	**81.50 ± 2.99**	74.00 ± 6.81 *	73.17 ± 7.68 *	70.50 ± 6.19 *	77.83 ± 5.21	56.33 ± 7.06 *	76.50 ± 5.90 *	70.33 ± 6.23 *
GSE116251	**90.50 ± 4.97**	84.50 ± 5.50 *	84.00 ± 8.76	74.00 ± 5.16 *	88.00 ± 4.83	62.00 ± 13.58 *	86.50 ± 5.30	78.00 ± 4.83 *
GSE142699	**100.00 ± 0.00**	93.33 ± 4.51 *	90.33 ± 5.02 *	97.50 ± 2.26 *	94.67 ± 3.58 *	97.33 ± 3.06 *	97.83 ± 2.84 *	99.33 ± 1.41
GSE158284	79.50 ± 5.99	76.00 ± 4.59	76.50 ± 6.26	77.50 ± 5.89	76.00 ± 6.58	77.00 ± 4.22	77.00 ± 10.06	**85.00 ± 7.45**
Ave	**89.49**	82.54	83.11	82.95	85.42	75.31	85.84	83.33
W/T/L		9/0/0	9/0/0	9/0/0	9/0/0	9/0/0	9/0/0	8/0/1

Bold: the highest specificity for the corresponding dataset. *: BIM-Ken statistically significant (at 0.05 level) wins or loses the corresponding method.

**Table 5:** The average performance of the defined modules in the independent validation dataset KIRC (Mean ± S.D.%).

Module id	Classification Accuracy Rate	Sensitivity	Specificity
M_1	97.10 ± 0.30	97.51 ± 0.39	95.71 ± 0.00
M_2	94.82 ± 0.32	96.35 ± 0.17	89.57 ± 1.79
M_3	97.33 ± 0.22	97.51 ± 0.34	96.71 ± 0.69
M_4	92.31 ± 0.24	95.15 ± 0.28	82.57 ± 0.60
M_5	97.11 ± 0.34	97.72 ± 0.22	95.00 ± 1.01
M_6	96.11 ± 0.35	96.63 ± 0.41	94.29 ± 1.17
M_7	87.49 ± 0.44	94.39 ± 0.35	63.71 ± 1.20

**Table 6:** The enriched representative pathway by the target genes of each identified module.

Module id	Pathway	*p*-Value
M_1	hsa04010:MAPK signaling pathway	2.45 × 10^−3^
M_2	hsa05211:Renal cell carcinoma	7.71 × 10^−4^
M_3	hsa05211:Renal cell carcinoma	2.15 × 10^−3^
M_4	hsa04350:TGF-beta signaling pathway	1.20 × 10^−3^
M_5	hsa05211:Renal cell carcinoma	4.36 × 10^−3^
M_6	hsa05211:Renal cell carcinoma	7.51 × 10^−6^
M_7	hsa04350:TGF-beta signaling pathway	8.72 × 10^−3^

## Data Availability

I have shared the link to public datasets in my manuscript.

## Supplementary Materials

The following supporting information can be downloaded at https://www.mdpi.com/article/10.3390/genes16080902/s1: Figure S1: The overview of BIM-Ken; Figure S2: Parameter analysis of the module number *k*; Table S1: Summary of key notations; Table S2: The miRNA information in identified modules.
