# Supplementary material for: BIM-Ken: Identifying Disease-Related miRNA Biomarkers Based on Knowledge-Enhanced Bio-Network
Source: Genes (Basel). 2025 Jul 28;16(8):902. doi: 10.3390/genes16080902 (PMC12386105; doi:10.3390/genes16080902)
Supplement: Supplementary file 1 [file genes-16-00902-s001.zip › genes-3761486-supplementary.pdf]

BIM-Ken: Identifying Disease-Related miRNA Biomarkers Based on Knowledge-Enhanced Bio-Network

Yanhui Zhang[1], Kunjie Dong[1], Wenli Sun[1], Zhenbo Gao[1], Jianjun Zhang[2], and Xiaohui Lin[1,*]

[1] School of Computer Science and Technology, Dalian University of Technology, Dalian 116024, China

[2] Department of Gastric Surgery, Cancer Hospital of Dalian University of Technology (Liaoning Cancer Hospital & Institute), Shenyang 110042, China

* Correspondence:

Prof. Xiaohui Lin, School of Computer Science and Technology, Dalian University of Technology, Dalian 116024, China. E-mail: datas@dlut.edu.cn.

The overview of BIM-Ken

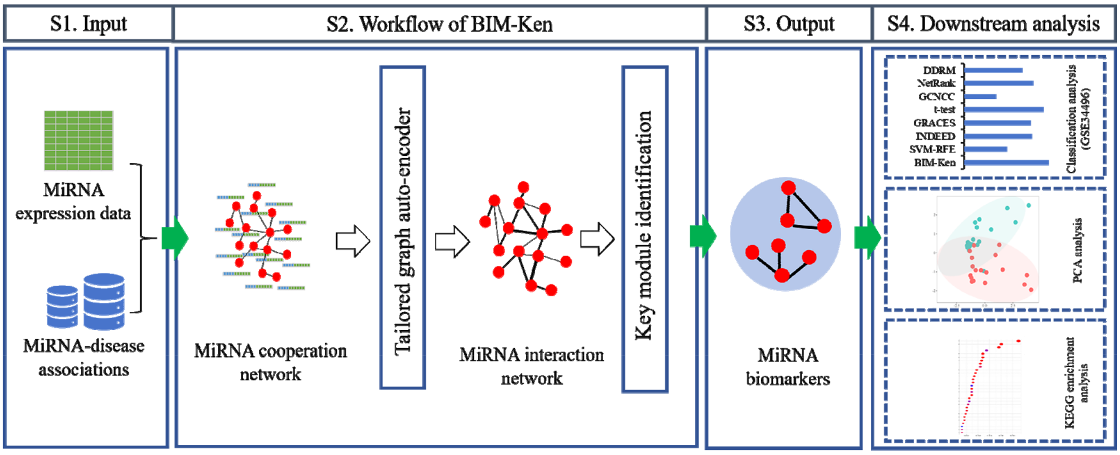

Figure S1. The overview of BIM-Ken.

Parameter analysis of the module number *k*

Since the number of modules may affect the performance of BIM-Ken, we analyzed the average classification accuracy rate of BIM-Ken over all datasets for the setting of module number *k* (*k* = 1, 3, 5, 7, 9). As shown in Figure S2, the average classification accuracy rate of BIM-Ken increased as *k* increased from 1 to 7, decreased as *k* increased from 7 to 9, and BIM-Ken achieved the highest average classification accuracy rate when *k* was 7. As the module number increases, more efficient information is introduced and the classification performance improves. If too many modules are selected, noise may also be introduced, which may degrade the classification performance. According to Figure S2, the module number (*k*) was set as 7 in this study.

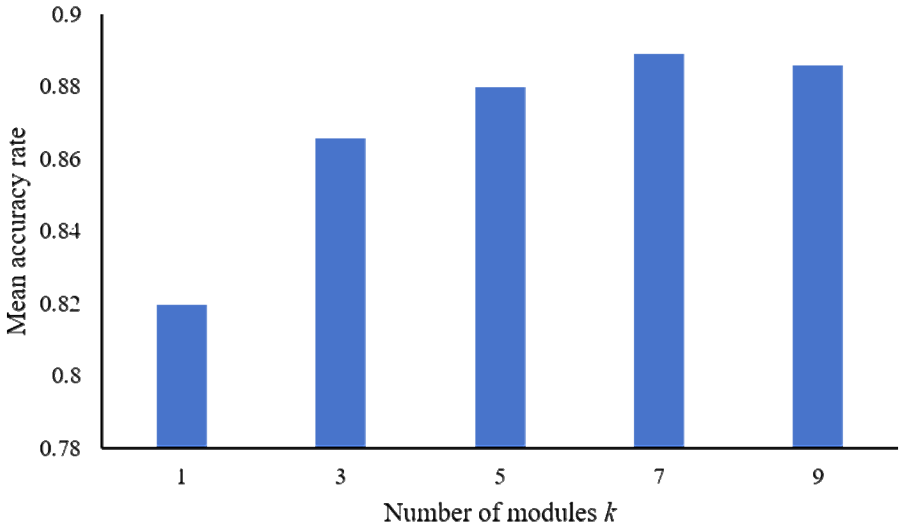

Figure S2. Parameter analysis of the module number *k*.

Summary of key notations

Table S1. Summary of key notations.

Symbols	Descriptions
S	The training sample set
F	The feature (miRNA) set
G_{co}	MiRNA cooperation network
X_{co}	Node attribute matrix
A_{co}	The adjacency matrix computed from G_{co}
Z	The node learned latent representations
A_{rec}	The reconstructed adjacency matrix
GS	Gaussian interaction profile kernel similarity matrix
G_{Net}	MiRNA interaction network

The miRNA information in identified modules

Table S2. The miRNA information in identified modules.

Module id	miRNAs
M_1#	hsa-miR-1537-3p; hsa-miR-335-5p; hsa-miR-155-5p; hsa-miR-197-5p
M_2#	hsa-miR-93-5p; hsa-miR-21-5p; hsa-miR-125a-3p; hsa-miR-127-3p
M_3#	hsa-miR-361-3p; hsa-miR-210-3p; hsa-miR-532-5p; hsa-miR-520a-5p; hsa-miR-503-5p; hsa-miR-342-3p
M_4#	hsa-miR-1260a; hsa-miR-592; hsa-miR-154-5p; hsa-miR-425-5p
M_5#	hsa-miR-27b-3p; hsa-miR-514a-3p; hsa-miR-1193; hsa-miR-455-5p; hsa-miR-382-5p; hsa-miR-374a-5p; hsa-miR-885-5p
M_6#	hsa-miR-222-3p; hsa-miR-15a-5p; hsa-miR-1-5p; hsa-miR-580-3p; hsa-miR-509-3p; hsa-miR-181a-5p
M_7#	hsa-miR-331-3p; hsa-miR-106b-5p; hsa-miR-98-3p

[1] #: the module identified by BIM-Ken is associated with renal cell carcinoma.
